# Water‐Induced Local Redox Reactions on Individual Ti_3_C_2_T*_x_* MXene Flakes in Aqueous Environment

**DOI:** 10.1002/anie.202520508

**Published:** 2025-12-04

**Authors:** Faidra Amargianou, Peer Bärmann, Namrata Sharma, Mailis Lounasvuori, Andreas Furchner, Ralfy Kenaz, Saptarshi Ghosh, Jan‐David Förster, Christopher Pöhlker, Markus Weigand, Tristan Petit

**Affiliations:** ^1^ Helmholtz‐Zentrum Berlin für Materialien und Energie GmbH Albert‐Einstein‐Straße 15 12489 Berlin Germany; ^2^ Faculty of Mathematics and Natural Sciences TU‐Berlin Hardenbergstraße 36 10623 Berlin Germany; ^3^ Racah Institute of Physics The Hebrew University of Jerusalem Jerusalem 9190401 Israel; ^4^ Multiphase Chemistry Department Max Planck Institute for Chemistry Hahn‐Meitner‐Weg 1 55128 Mainz Germany; ^5^ Atmospheric Microphysics Department Leibniz Institute for Tropospheric Research Leipzig Germany

**Keywords:** Confined water, In situ, MXenes, Redox reactions, X‐ray microscopy

## Abstract

Water at an interface, confined in nanopores or between layers exhibits unique structural and dynamic properties that differ significantly from bulk water. In layered 2D materials such as MXenes, intercalated water is believed to affect their surface chemistry by inducing local oxidation and contribute to redox processes during electrochemical cycling. However, the chemical nature of confined water and its interaction with MXene surface chemistry remains unclear. Here, we employ scanning transmission X‐ray microscopy (STXM) to investigate in situ the chemical interaction of water in individual Ti_3_C_2_T*_x_* MXene flakes in humid and aqueous environments with ∼50 nm spatial resolution. At the oxygen K‐edge, we uncover that water trapped in pockets and wrinkles in few‐layered MXene flakes has a different hydrogen bonding compared to water confined in the MXene interlayer spacing. We also reveal water‐induced local redox reactions of Ti atoms non‐uniformly distributed on the MXene flake upon interaction with liquid water and alkali ion neutral electrolytes, which are partly reversible upon exposure to an acidic electrolyte.

## Graphical abstract

The local chemistry of individual Ti_3_C_2_T*_x_* MXene flakes and confined water are investigated by in situ Scanning Transmission X‐ray Microscopy (STXM) in aqueous environment. We reveal water‐induced redox reactions non‐uniformly distributed over the MXene flakes upon sequential exposure to acidic and neutral aqueous solution containing alkali cations.

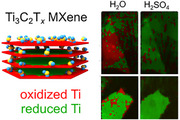

## Introduction

Understanding the interaction between water molecules, protons and cations with electrode surfaces is essential for many applications, including electrochemical energy storage, electrocatalysis and desalination.^[^ ^1^, ^2^, ^3^, ^4^ ^]^ In layered 2D materials, such as MXenes, consisting of transition metal carbide and nitrides, confinement effects in the interlayer spacing may provide new avenues to modulate the pseudocapacitive behavior of the host materials^[^ ^5^ ^]^ or modulate the activity or selectivity of electrochemical reactions.^[^ ^6^, ^7^ ^]^ Moreover, confined water plays a role in the chemical stability of MXene. While Ti_3_C_2_T*_x_* MXene dispersed in water may be prone to hydrolysis within a few hours,^[^ ^8^ ^]^ Ti_3_C_2_T*_x_* MXene films have been reported to remain stable over several years.^[^ ^9^ ^]^ It was recently reported that water confined between MXene flakes may be a source of oxidation.^[^ ^10^ ^]^

As a result of the strong influence water has on the properties of MXenes, water intercalation within MXene interlayer spacing has been studied by several analytical techniques. The pre‐intercalation of cations was found to affect the intercalated water layer. A stepwise expansion of Ti_3_C_2_T*_x_* MXene with humidity related to cation hydration enthalpy was revealed by X‐ray diffraction (XRD).^[^ ^11^ ^]^ Thermogravimetric analysis,^[^ ^11^ ^]^ nuclear magnetic resonance^[^ ^12^ ^]^ and infrared spectroscopy^[^ ^13^ ^]^ have revealed that both bulk‐like and confined water was present in MXene films, but all these techniques average the signal recorded over large ensemble of MXene flakes. The local distribution of these different water phases and the interplay with MXene surface chemistry remain largely unexplored because it requires the characterization of single MXene flakes.

We have recently shown that the chemical sensitivity of X‐ray absorption spectroscopy (XAS) at the oxygen K edge, combined with a high spatial resolution, is ideally suited to revealing local changes of intercalated water within MXene films.^[^ ^14^ ^]^ In addition, XAS at the Ti L‐edge is sensitive to chemical change of the Ti bonding environment, which was found to depend on cation intercalation.^[^ ^15^ ^]^ However, the interplay between confined water and intercalated species and the MXene surface chemistry remains unresolved, as conventional techniques average over many flakes and do not distinguish between different local environments such as basal planes or edges. Previously, the surface chemistry of pristine Ti_3_C_2_T*_x_* MXene was investigated in vacuum using scanning transmission X‐ray microscopy (STXM).^[^ ^16^ ^]^ However, spatially resolved XAS under different environmental conditions is still lacking.

**Figure 1:**
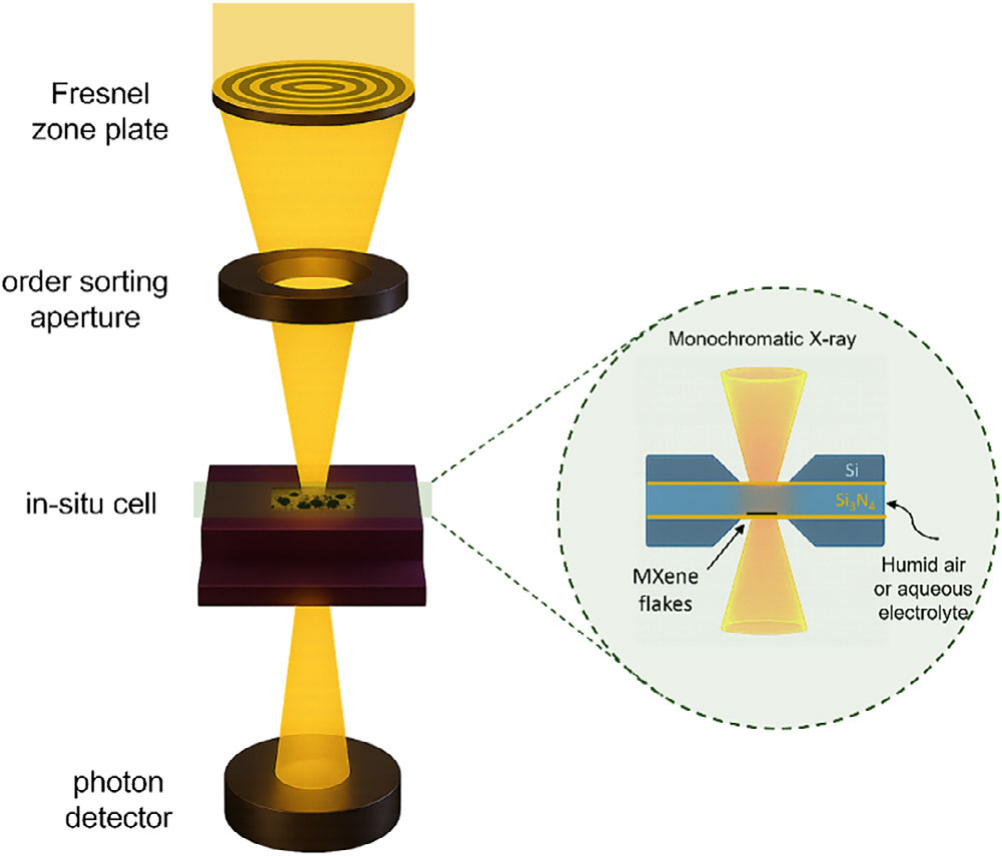
(a) Schematic of the in situ STXM setup with a Fresnel zone plate for X‐ray focusing, an order‐sorting aperture allowing only 1^st^‐order diffracted X‐rays to reach the sample, and a photon detector capturing transmitted X‐rays. The microscope is coupled with an in situ liquid cell. (b) Cross section of the in situ cell allowing exposure of MXene flakes to a layer (<10 µm) of aqueous electrolyte or humid air.

In this work, we employ in situ STXM to image the local interaction of water with individual Ti_3_C_2_T*_x_* MXene flakes under humid air and in aqueous environment (Figure 1). Nanoscale imaging with STXM allows the resolution of local water bonding and the correlation with the chemical environment with variations in MXene flake thickness and geometry, that remain inaccessible to bulk techniques. The nature of the water hydrogen bonding in wrinkles, at basal planes and edges of individual MXene flakes is probed at the O K‐edge. The role of the aqueous medium on the MXene surface chemistry is further investigated at the Ti L‐edge in acidic electrolyte, as well as diluted neutral electrolytes containing alkali cations (Li^+^, Na^+^, K^+^). We reveal thickness‐dependent increase of the oxidation state of the Ti atoms in the Ti_3_C_2_T*_x_* MXene flakes, which can be reversible or not depending on the conditions.

## Experimental Section

### MXene Synthesis

The synthesis of multilayered Ti_3_C_2_T_x_ MXene flakes was performed following the procedure proposed by Mathis et al.^[^ ^17^ ^]^ Briefly, one gram of MAX phase was slowly added to a mixture of 6 mL H_2_O, 12 mL (37 wt.%) HCl, and 2 mL (49 wt.%) HF in a vented polytetrafluoroethylene bottle. The solution was stirred at 300 rpm for 24 h at 35 °C. The resulting multilayered MXene was then centrifuged at 2500 relative centrifugal force for 5 min in a 175 mL tube in ultrapure water, and the clear supernatant was decanted. This centrifugation and decantation process was repeated until the supernatant reached a pH of ≈ 6. For delamination, multilayer MXene was stirred in LiCl solution, and after centrifugation the supernatant was decanted. After refilling with water, MXene redispersion was done multiple times until a transparent supernatant indicated a low MXene concentration. LiCl concentration decrease led to spontaneous MXene delamination, forming a stable, darker colloidal solution. For the experiments in humid air, the MXene is washed two times with 4 M HCl, three times with distilled water and heated to 80 °C to remove intercalated water before loading the sample into the microscope.^[^ ^3^ ^]^ For the in situ STXM measurements, the MXene same was freshly synthesized (within a week) before the experiments. A MXene sample, prepared with similar conditions and stored in closed deaerated vial in the fridge at 5 °C, was used for observing long‐term oxidation processes.

### STXM imaging

The STXM measurements were performed at the “MAXYMUS” and “MYSTIIC” microscope endstation at HZB/BESSY II. The transmission X‐ray flux was recorded using a photomultiplier tube. Two different STXM holders were used for measurement in humid air or liquid conditions. For humid conditions, a special microreactor system for in situ STXM under high humidities and low temperatures was used.^[^ ^18^ ^]^ The in situ microreactor system allows STXM characterization in relative humidity (RH) ranging from 5 to 90 (± 5) %.^[^ ^18^, ^19^ ^]^ The humidity holder is used to investigate the confinement and intercalation of water molecules in Ti_3_C_2_T*_x_* MXene flakes during in situ exposure to controlled humidity. The sample was exposed to the different humidity for at least an hour before STXM measurements. Another in situ holder designed and fabricated by NORCADA was used for STXM imaging in liquid media, enabling a smaller spacing (< 10 µm) between the two silicon nitride windows onto which Ti_3_C_2_T*_x_* MXene flakes were deposited by drop casting. The holder is made of a PEEK body to allow the use of corrosive electrolytes such as sulfuric acid. The concentration of the acidic (H_2_SO_4_) and neutral (LiCl, KCl, NaCl) electrolytes was set to 0.1 M to avoid significant attenuation of the soft X‐rays by the ions upon switching the electrolytes that would not allow direct comparison to the deionized water. The MXene flakes were sequentially exposed to different aqueous electrolytes. The exposure time to the electrolytes before the STXM measurement was at least an hour to reach steady state conditions.

The STXM data are collected with point scanning and image scanning methods. For the humidity measurements, images are acquired at the energy ranges from 530 to 550 eV (O K‐edge) and from 450 to 475 eV (Ti L‐edge) with energy steps of 0.2 eV. For the liquid measurements we acquire images at maximum absorption peaks at the Ti L‐edge (457.8, 459.6, 463.2 and 465.3 eV) with a pixel size of 50 nm and a dwell time of 2 ms to avoid prolonged X‐ray exposure of the sample. However, point scans with defocus of 1 µm spot size on selected areas of the flakes are acquired at Ti L‐edge (450 to 475 eV with an energy step of 0.15 eV). Considering a dwell time of 500 ms and the time to move the monochromator, the duration of one point scan at the Ti L‐edge is estimated to ∼2 min, which leads to local oxidation as discussed in Figure S8.

### Data Analysis

For the full energy stack at the O K‐edge, k‐means clustering is performed to identify two regions (pristine flakes and pockets). The colored images in Figure 4b–e are created by identifying regions in the MXene flakes of increased Ti oxidation state by using the Ti L_2_ *e_g_ / t_2g_* peak intensity ratio as a metric. Regions with a peak intensity ratio greater than 0.98 are defined as areas of increased Ti oxidation state (threshold method). This threshold was chosen based on the ratio of the XAS obtained for MXene exposed to KCl, NaCl, and LiCl aqueous solutions (Ti L_2_ *e_g_ / t_2g_* peak ratio > 0.98), and H_2_SO_4_ (Ti L_2_ *e_g_ / t_2g_* peak ratio < 0.98), and averaged over the whole MXene flakes. To suppress shot noise, a median filter was applied to the Ti L_2_ *e_g_ / t_2g_* contrast maps in Figure 4b–e, replacing each pixel with the median of its local neighborhood. However, to generate the contrast map in Figure S8, an autoencoder was used for denoising, as the full Ti L‐edge stack used in this case had a lower spatial resolution of 1 µm, compared to 50 nm for the other images, while maintaining the same dwell time per pixel (2 ms), resulting in a lower signal‐to‐noise ratio. In Figure 3c a moving average filter with a window size of 5 is applied by replacing each point with the average of itself and its two nearest neighbors on each side.

The ex‐situ STXM measurements consist of point scans and images at the O K‐edge and Ti L‐edge. The RGB image at the O K‐edge is created by acquiring images at absorption peaks (531.4, 534.4 and 537.4 eV), characteristic of different oxygen species. The threshold method described above is used for the colored image of this flake at the Ti L‐edge. The data analysis is performed with custom Python scripts. Image sequences, measured at the Ti L‐ and O K‐edge, were aligned with the software aXis2000.

### Scanning Electron Microscopy (SEM)

**Figure 2:**
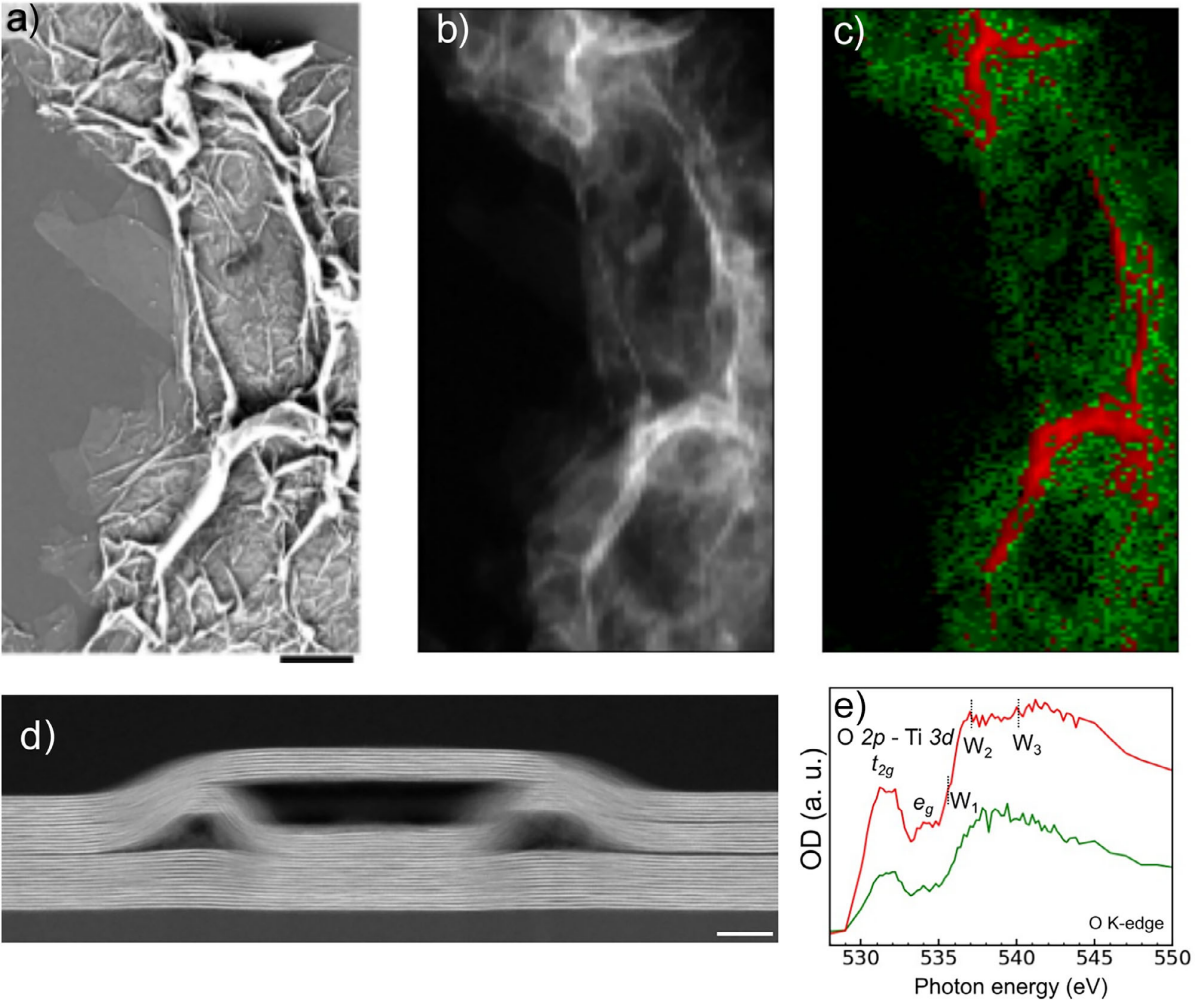
Chemical imaging of water trapped in a crumpled Ti_3_C_2_T_x_ MXene film. a) Correlative SEM image and b) STXM image, averaged at the O K‐edge (528‐550 eV), of the MXene film. Scalebar: 2 µm. c) Composite image with bulk‐like water adsorbed on MXene overlapping flakes (green) and trapped water in large wrinkles (red) measured in dry air obtained by clustering based on the XAS data shown in e. d) STEM image of a wrinkled 31‐layered Ti_3_C_2_T_x_ MXene flake with nanometer‐scaled pockets. Scale bar: 20 nm. e) XAS spectra at the O K‐edge of water trapped in MXene wrinkles and water on top of MXene flakes as identified from clustering. The relative humidity is 5 (± 5) % and the temperature is 20 °C.

The image in Figure 2a is acquired by a Zeiss Merlin SEM at Helmholtz Zentrum Berlin, using secondary electron detection at 3.00 kV accelerating voltage, 150 pA probe current, 800 V for the energy Selective Backscatter detectors’ grid, and 6.6 mm working distance. The pressure in the SEM chamber was ∼2 × 10^−6^ mbar.

### Scanning Transmission Electron Microscopy (STEM)

Cross‐section lamellas, approximately 100 nm thick, were prepared from selected MXene flakes using a focused ion beam instrument (FIB, Helios Nanolab 460F1 Lite, Thermo Fisher Scientific). These lamellas were then transferred onto TEM grids with the help of a micromanipulator. Imaging was carried out using a scanning transmission electron microscope (STEM, Themis Z G3, Thermo Fisher Scientific) operated at 300 kV. A high‐angle annular dark‐field (HAADF) detector was used for contrast. The resulting STEM images were analyzed using the Velox software (Thermo Fisher Scientific).

## Results

### Water Confinement in Thin Ti_3_C_2_T_x_ MXene Films

Different water phases may be found based on the various chemical environment observed in few‐layered MXene flakes. While isolated and 2–3 layer‐thin water films may be found in the interlayer spacing, water molecules found in wrinkles or pockets within MXene films may have more extensive hydrogen bonding. Adsorbed water on top of the flake is also expected in humid environment, which hydrogen bonding must differ from confined water. The water signature was first characterized using in situ STXM in a crumpled film of few‐layered Ti_3_C_2_T_x_ MXene flakes exposed to humid air with RH ranging from 5% to 70% (± 5%). SEM (Figure 2a) and STXM image of Ti_3_C_2_T_x_ MXene (Figure 2b) show micrometer‐long wrinkles, next to single‐ and few‐layered overlapping MXene flakes in which water may be trapped. The thickness of the Ti_3_C_2_T_x_ MXene flakes can be estimated from the X‐ray optical density at the Ti L‐edge.^[^ ^16^ ^]^ The thickest area of the films is in the order of ∼20 MXene layers (Figure S1). In addition, STEM images recorded on similarly prepared Ti_3_C_2_T_x_ MXene flakes exhibit sub‐nanometer interlayer spacing as well as pockets of 20–80 nm in wrinkled few‐layered MXene flakes (Figure 2d). All these different gaps and pores in the MXene film, ranging from sub‐nanometer to micrometer sizes, may lead to different types of water hydrogen bonding networks, which are probed using XAS at the O K‐edge (Figure 2c,e). The contributions from the O 2p orbitals of oxygen functional groups that hybridize with the Ti *3d* orbitals in the MXene, with *t_2g_* and *e_g_* configurations, are visible at 531.4 eV and 534.4 eV. Above 535 eV, contributions from water molecules overlap with the X‐ray absorption of the MXene O‐termination (O *2p* – Ti *4sp* orbitals).^[^ ^16^ ^]^

The typical pre‐, main‐ and post‐edge regions related to hydrogen‐bonded water molecules are detected at 535.1 eV (W_1_), 536.8 eV (W_2_) and 540 eV (W_3_), respectively, with different intensities in the large wrinkles and the thinner MXene flakes (Figure 2c). The broad X‐ray absorption band around 540 eV of oxygen‐containing functional groups^[^ ^14^, ^16^ ^]^ may contribute to the W3 component. The variation of these three components provides information on the water hydrogen bonding network that differs between large wrinkles and on the rest of the MXene flakes.^[^ ^20^, ^21^ ^]^ In large wrinkles, the higher W_3_ contributions suggests a long‐range ordering of water molecules.^[^ ^22^ ^]^ Increasing the relative humidity from 5% to 70% (Figure S2) leads to an increase in the XAS signal of the W_2_ and W_3_ components that agree with an extended hydrogen bonding network similar to bulk‐like water. Within crumpled few‐layered MXene flakes, the W_3_ feature appears to be reduced compared to large wrinkles. The water molecules detected in thinner overlapping MXene flakes exhibit an enhanced mid‐range ordering (W_2_), like interfacial water detected in nanodiamond dispersions.^[^ ^23^ ^]^ This type of hydrogen‐bonding may be related to water adsorbed on the top of the MXene flake or water trapped within the pockets between overlapping MXene flakes that are observed by STEM (Figure 2d).

**Figure 3:**
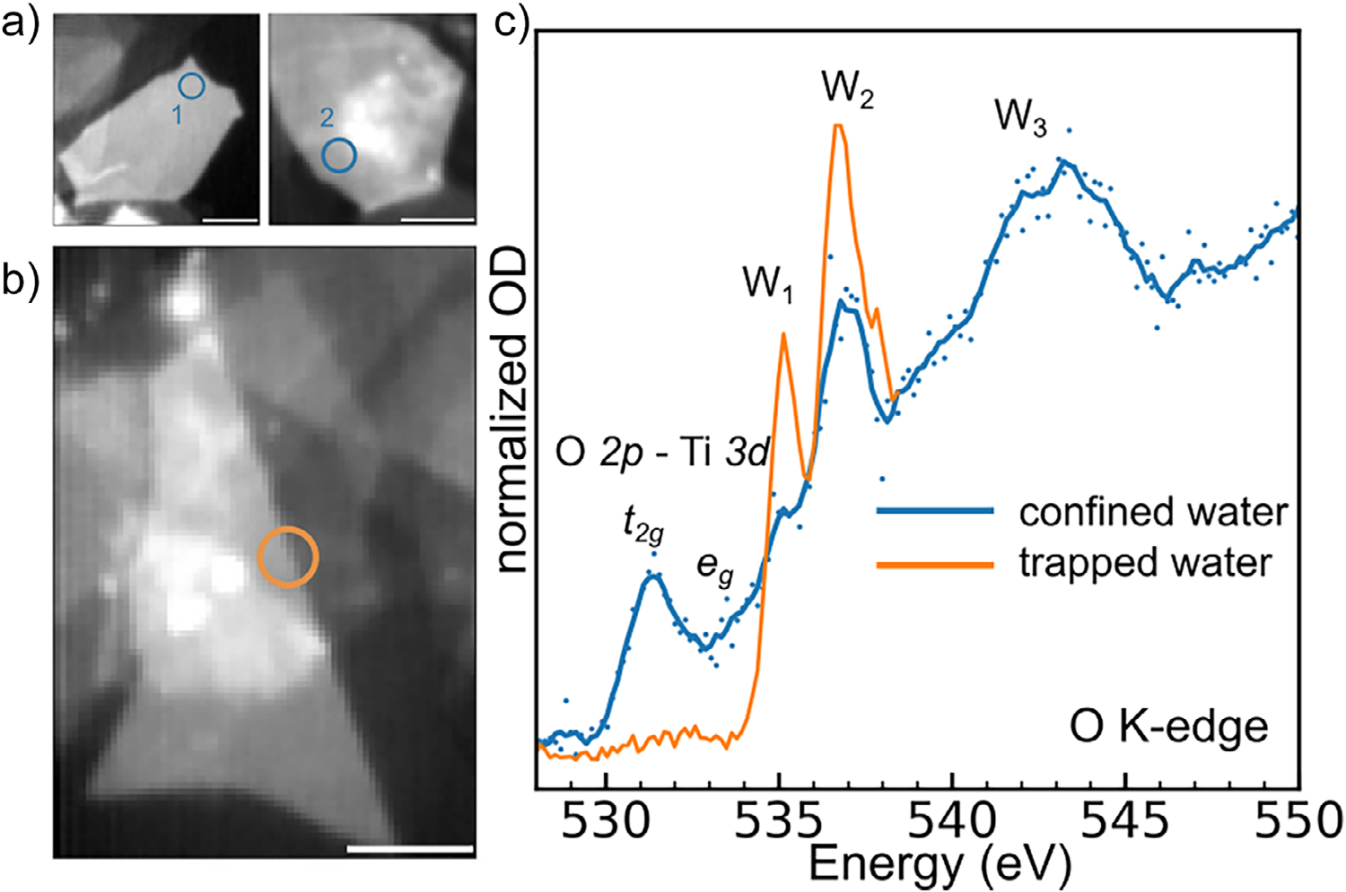
Water phases in isolated few‐layered Ti_3_C_2_T*_x_* MXene flakes. STXM images at Ti L‐edge for a) isolated few‐layered MXene flakes and b) overlapping few‐layered MXene flakes. Scale bar: 1 µm. c) XAS spectra at the O K‐edge, acquired for water trapped between overlapping MXene flakes (orange) and confined within the basal plane of a few‐layered Ti_3_C_2_T_x_ MXene flake (blue). The XAS of confined water is acquired by dividing the XAS acquired on the 20‐layered MXene flake (region 2) by the one acquired on the 10‐layered flake (region 1) to reduce the contribution of water phases other than the water confined in the interlayer (OD: optical density).

Compared to large wrinkles, spatial confinement within the MXene interlayer spacing (<1 nm) may lead to a very different hydrogen bonding environment compared to bulk water as suggested by previous work.^[^ ^13^, ^14^ ^]^ Given that the STXM measurements are performed in transmission, the contribution of water confined in the MXene interlayer is screened by the bulk water observed in the crumpled films. Therefore, isolated individual few‐layered MXene flakes with thicknesses below 20 layers as determined by their X‐ray absorption at the Ti L‐edge (Figure S3) were investigated to probe confined water (Figure 3a,b). The MXene flakes were exposed to liquid water and then dried under nitrogen flow until most of the bulk water was removed. We assumed that the confined water is not fully removed by the nitrogen flow at room temperature as shown by our previous work using in situ FTIR.^[^ ^3^ ^]^ An annealing in vacuum at 600 °C would be necessary to fully remove intercalated water,^[^ ^10^ ^]^ which is not possible in our in situ cell.

The XAS at the O K‐edge recorded on few‐layered MXene flakes (Figure 3c) differs significantly from the previously observed water phases (Figure 2e). The W_1_ and W_2_ components appear sharp, resembling the XAS reported for isolated water molecules^[^ ^20^, ^21^ ^]^ or confined within nanoporous carbon materials.^[^ ^19^ ^]^ These peaks are clearly associated to water molecules because they are also detected on areas where no significant contribution from the transition to *t_2g_* states from the oxygen surface groups are observed (Figure 3b,c). In this region, most of the X‐ray absorption from oxygen may originate from water molecules trapped in pockets formed between MXene flakes. On the other hand, the difference XAS, obtained by dividing the XAS of ∼20 layered MXenes by the one recorded for 10 layered flakes, allows us to extract the signature of water molecules confined in the MXene interlayer (Figure 3a,c). In this case, the *t_2g_* and *e_g_* contributions from the MXene surface groups are clearly visible. Water confined within the MXene interlayer presents a reduced W_1_ peak, a well‐defined W_3_ peak and broader lines compared to the gas‐like water, resembling the signature of nanoconfined water recently reported.^[^ ^14^ ^]^

**Figure 4:**
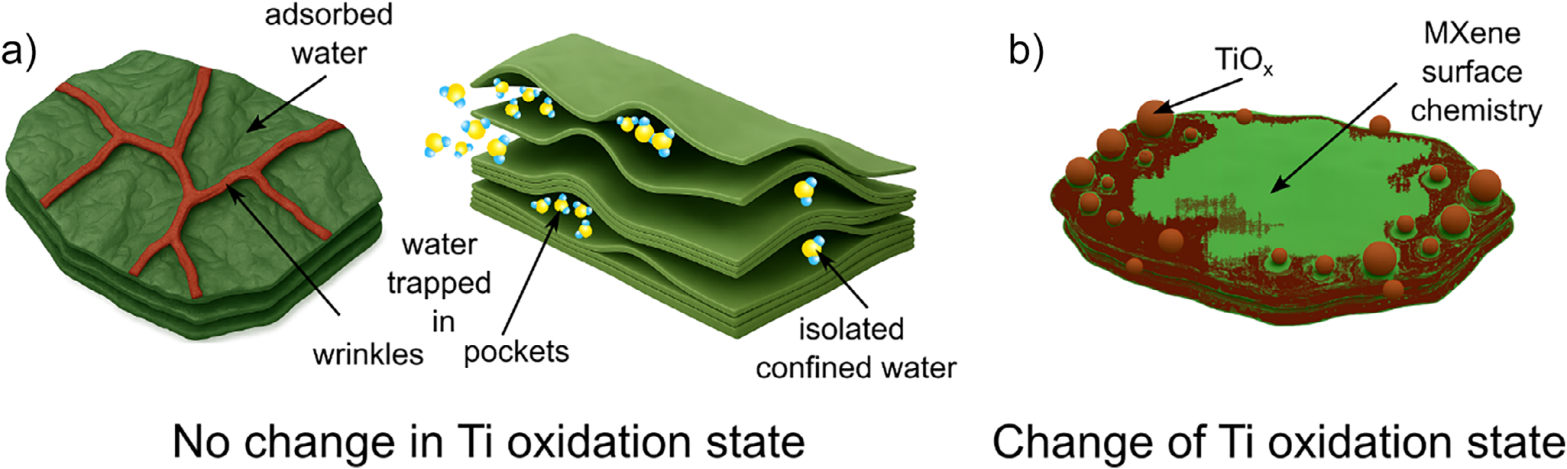
Schematic view of the different origins of oxygen from water phases a) and MXene flakes b). Trapped and adsorbed water do not induce change of the Ti oxidation state while change of MXene surface chemistry and formation of titanium oxide particles do induce a change of the Ti oxidation state.

Thanks to the high chemical sensitivity and spatial resolution of STXM, we have identified four different types of water phases with different hydrogen bonding environments, which are schematically shown in Figure 4a: (i) confined water molecules within the MXene interlayer, (ii) gas‐like water probably found in pockets between overlapping MXene flakes, (iii) trapped water in nanosized wrinkles, and (iv) bulk‐like water in large wrinkles and adsorbed on the surface of the flake.

### Redox Reaction on Ti_3_C_2_T_x_ MXene Flakes in Aqueous Environment

**Figure 5:**
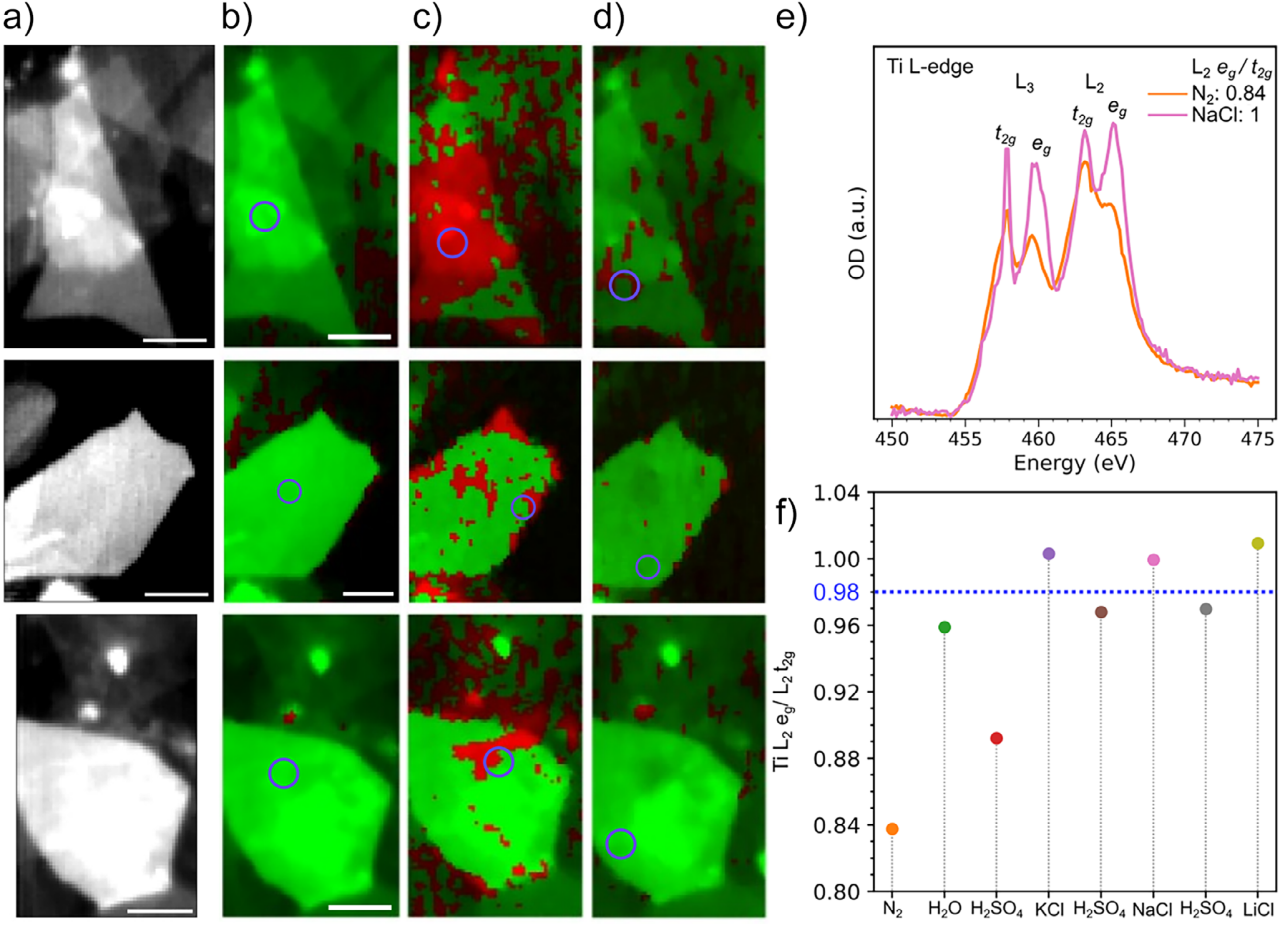
Redox reaction on Ti_3_C_2_T_x_ MXene flakes in aqueous electrolytes. a) STXM images of MXene flakes with thickness 1–10 layers (top), 10 layers (middle) and 20 layers (bottom), averaged at Ti L‐edge and exposed to N_2_ gas. Scalebar: 1 µm. STXM images at Ti L‐edge during exposure to b) N_2_ gas, c) H_2_O, d) 0.1 M H_2_SO_4_, colored based on L_2_ *e_g_* to L_2_ *t_2g_* peak intensity ratio, reflecting the oxidation state of Ti atoms. Green (red) regions correspond to ratio lower (higher) than 0.98. e) XAS at Ti L‐edge for the 20‐layered Ti_3_C_2_T_x_ MXene flake during exposure to N_2_ gas and 0.1 M NaCl aqueous electrolyte. f) XAS Ti L_2_ *e_g_* to *t_2g_* peak intensity ratio was evaluated for the 20‐layered flake during sequential exposure to N_2_ gas, H_2_O, H_2_SO_4_, KCl, H_2_SO_4_, NaCl, H_2_SO_4_ and LiCl (0.1 M for all electrolytes). The blue circles on the flakes indicate the area where the point scan XAS spectra were collected.

The MXene surface chemistry, monitored at the Ti L‐edge, does not seem to be altered on few‐layered Ti_3_C_2_T_x_ MXene flakes by short term exposure to humid air (Figure S1). However, more dramatic changes of the MXene surface chemistry may be induced by exposure to aqueous environment, as well as by the presence of cations (K^+^, Na^+^, or Li^+^) in the aqueous environment.^[^ ^24^ ^]^ Three few‐layered Ti_3_C_2_T_x_ MXene flakes, with thickness ranging from 1 to 20 layers, are imaged using STXM at the Ti L‐edge under nitrogen flow (Figure 5a,b) and various aqueous environments (Figure 5c,d and Figure S4). The morphology of all MXene flakes remains intact but the chemical bonding of Ti atoms changes dramatically in the different aqueous environments (Figure 5e,f). The XAS Ti L_2_ *e_g_/t_2g_* peak intensity ratio is used as a qualitative indicator of the Ti oxidation state. In an octahedral crystal field, the 3*d* orbitals split into lower‐energy *t_2g_* and higher‐energy *e_g_* levels. As the oxidation state of titanium increases, the number of 3*d* electrons decreases, leading to more unoccupied *d* states. This results in increased absorption intensity, particularly at the energy related to the transitions related to *e_g_* states, which is more sensitive to changes in oxidation due to its stronger interaction with surrounding ligands. For example, the XAS spectrum of a 20‐layered flake under N_2_ gas shows a ratio of 0.84 (Figure 5e), characteristic of a nearly pristine state.^[^ ^16^ ^]^ Upon exposure to 0.1 M NaCl, the ratio increases to 1, indicating substantial surface oxidation (Figure 5e).

The chemical imaging of the Ti oxidation state in various aqueous environments is achieved by setting a threshold value for the XAS Ti L_2_ e_g_/t_2g_ peak of 0.98 to distinguish between reduced and oxidized regions within the Ti_3_C_2_T_x_ MXene flakes (Figure 5b,e). An increased Ti oxidation state was observed upon exposure to water (Figure 5c), especially for the thin (1–10 layers) MXene flakes. In this case, most of the MXene basal planes are oxidized, while discrete regions, mostly close to the edges, are oxidized for thicker flakes. Interestingly, this oxidation seems reversible upon exposure to 0.1 M H_2_SO_4_ on most of the surface of the MXene flakes (Figure 5d). The increased oxidation state observed in overlapping 1–10 layered flakes may be attributed to higher delamination, allowing easier water access to both the basal plane and edges. In contrast, the 20‐layered MXene shows oxidation primarily at the edges, as interlayer Van der Waals interactions may reduce water penetration into the basal plane. Nevertheless, water intercalation is confirmed by the O K‐edge XAS supporting the presence of confined water between the layers. Exposure to an acidic electrolyte may remove free water while protonating the MXene surface, which reduces the averaged Ti oxidation state.^[^ ^15^ ^]^ These experimental observations are in line with recent theoretical work on water‐induced degradation of Ti_3_C_2_T_x_ MXene basal planes.^[^ ^25^ ^]^ While an initial stage involves a reversible water adsorption on the O‐terminated sites, it further evolves in a non‐reversible adsorption leading to the pull out of surface Ti atoms that is then coordinated to four oxygen molecules, that would correspond to a 4 + oxidation state. The non‐uniform oxidation of the MXene flake may be related to a mixed O/OH/F surface termination after wet chemical etching. While surface chemistry is known to be non‐uniform at the atomistic scale^[^ ^26^ ^]^ our results suggest that patches of predominantly O‐terminated groups, more prone to water‐induced reactions, are found in the range of few hundreds of nanometers. Edge‐induced degradation, eventually leading to MXene hydrolysis,^[^ ^27^ ^]^ is not observed here as we do not see any reduction of the MXene flake size during the measurement. Nevertheless, the increase of the Ti oxidation on the edge may constitute the initial stage of hydrolysis process that occurs on much longer timescale.^[^ ^28^ ^]^

Cation intercalation was also previously shown to induce an oxidation of the Ti atoms in MXene flakes.^[^ ^15^, ^29^ ^]^ MXene flakes were therefore exposed sequentially to a series of neutral aqueous solutions containing alkali cations (0.1 M KCl, NaCl and LiCl), alternating with H_2_SO_4_ to observe the role of cations in the MXene surface oxidation directly in liquid medium. For each electrolyte, an XAS Ti L‐edge spectrum was acquired as a point scan (Figure S5) as well as a corresponding STXM image (Figure S4). The Ti L_2_ e_g_/t_2g_ peak ratio extracted from the point scans are shown in Figure 5e. The reversible water‐induced oxidation, with a ratio increase from 0.84 to ∼0.96 upon exposure to water, is clearly visible. After H_2_SO_4_ exposure, the ratio decreases to 0.89, due to the reduction through surface protonation. When exposed sequentially to neutral cationic electrolytes (K^+^, Na^+^, Li^+^), the ratio increases again, with values approaching 1, consistent with Ti oxidation stronger than observed with pure water. For each cycle, the Ti oxidation is partially reversible upon subsequent acid exposure. However, the ratio does not recover to its original value in pure water, highlighting that the remaining cations may also contribute to local oxidation. This may be related to the passivation of defect sites, which has been recently reported on similarly prepared Ti_3_C_2_T_x_ MXenes.^[^ ^30^ ^]^ Since the electrolytes are replaced sequentially to image the same MXene flakes, it is not possible to compare the oxidation potential of each cations, as observed in our previous work performed on thick MXene films.^[^ ^15^ ^]^ In the future, parallel experiments with MXene flakes exposed to only one type of ion, recorded with high time‐resolution, will allow a dynamic understanding of intercalation processes in MXenes. This reversible oxidation‐reduction of the Ti atoms, which is well distributed over the full area of the MXene flakes (Figure S4), highlights that redox‐active sites are found over the whole few‐layered Ti_3_C_2_T_x_ MXene flakes. Note that X‐ray beam induced oxidation of the Ti atoms in the MXene flakes was also evidenced for point scan XAS measurements, indicated with blue circles on Figure 5. Indeed, chemical imaging reveals a more prominent oxidation close to the region selected for point scan imaging (see Figures S6 and S7). This oxidation may be related to the radiolytic species generated by prolongated exposure to the X‐ray beam. Note that radiolytic effects are generally less intense with soft X‐ray compared to electron beam, especially at the Ti L‐edge because the core levels of oxygen atoms are not directly excited. X‐ray induced radiolytic species can be used to study local chemical oxidation at the sub‐flake level,^[^ ^31^ ^]^ but they can also be avoided using STXM imaging with fast raster scanning, that will therefore be used for future work on electrochemically‐induced redox processes. While the X‐ray induced oxidation does not change the qualitative description of the reversible oxidation/reduction of the Ti atoms, a quantitative analysis is not possible at this stage. Note that the XAS Ti L_2_ *e_g_* to *t_2g_* peak intensity ratio also depends on the number of layers (Figure S3). Future STXM quantitative analysis must consider these parameters to enable a reliable comparison.

### Defect‐Induced Oxidation of Ti_3_C_2_T*_x_* MXene Flakes

**Figure 6:**
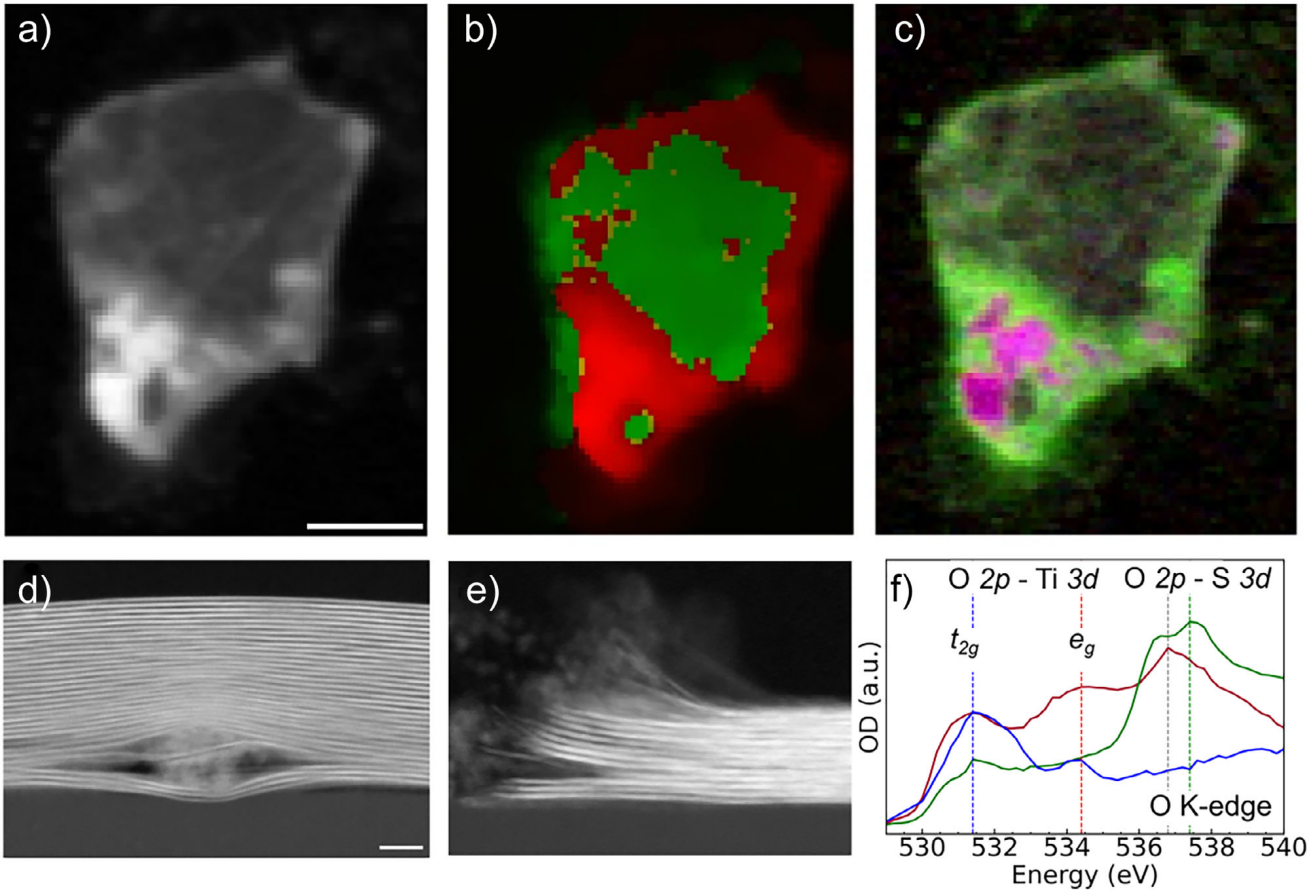
Ex situ imaging of Ti_3_C_2_T*_x_* MXene flake exposed to 0.1 M sulfuric acid in vacuum. a) STXM image of MXene, averaged over the energies 528, 531.4, 534.4 and 537.4 eV. Scale bar: 2 µm. b) STXM image at the Ti L‐edge with Ti L_2_ *e_g_ t_2g_* peak intensity ratio > 0.98 (red) and < 0.98 (green). c) Chemical mapping created by STXM images at 531.4 (blue), 534.4 (red) and 537.4 (green) eV. STEM images of d) TiO_2_ nanoparticles trapped between Ti_3_C_2_T*_x_* MXene layers, d) in a 31‐layer flake, and e) the edge of a 13‐layer flake. Scale bar: 10 nm. f) XAS spectra at O K‐edge of pristine MXene (blue), oxidized MXene (red) and MXene exposed to H_2_SO_4_ (green).

Το investigate the nature of oxidation induced by long‐term storage in water,^[^ ^32^ ^]^ The local surface chemistry of an individual Ti_3_C_2_T*_x_* MXene flake was investigated by STXM at the O K‐edge and Ti L‐edge (Figure 6). A 15‐layered Ti_3_C_2_T*_x_* MXene flake, shown in Figure 6a–c, was exposed to 0.1 M H_2_SO_4_ to investigate only irreversible oxidation. The XAS spectrum at the O K‐edge of pristine Ti_3_C_2_T*_x_* MXene in Figure 6f consists of two main peaks at 531.4 eV and 534.4 eV, noted as *t_2g_* and *e_g_* respectively, which are related to the interaction of O 2p antibonding molecular orbitals with the Ti 3d orbitals. After exposure to 0.1 M H_2_SO_4_, an additional strong absorption band at 537.4 eV appears, related to the O 2p orbitals of sulfate ions.^[^ ^30^ ^]^ A composite image (Figure 6c), constructed from STXM images recorded at 531.4 (blue), 534.4 (red) and 537.4 (green) eV, reveals a non‐uniform oxygen bonding throughout the MXene flake.

To distinguish between oxygen atoms associated with the MXene surface terminations and intercalated/adsorbed oxygen‐containing molecules, the MXene flake was further imaged at the Ti L‐edge (Figure 6b). As previously described, the chemical imaging of the MXene surface chemistry is achieved using the Ti L_2_ *e_g_ / t_2g_* peak intensity ratio as a measure of the local oxidation state of the Ti atoms. Even if there is a correlation of reduced Ti oxidation state (Figure 5b) and oxygen from sulfate ions (Figure 5c), the exposure of flakes to H_2_SO_4_ does not prevent the existence of oxidized regions. A higher *e_g_* component at both the oxygen and the titanium STXM images relates to the more oxidized regions. Higher oxidation at the edges is observed, consistent with our previous observation with X‐PEEM.^[^ ^29^ ^]^ In addition, localized areas with increased oxygen content correlate well with an increased Ti oxidation, which may indicate the local conversion of MXene into TiO_2_ nanoparticles. Compared to STEM cross‐section of a Ti_3_C_2_T*_x_* MXene flake prepared with a similar synthesis, such titanium oxide nanoparticles located between MXene layers were also evidenced (Figure 6d). Furthermore, clear distortions in the first 30 nm close to the edges are visible (Figure 6e). The initial Ti oxidation process that was revealed by in situ measurements in aqueous electrolyte therefore evolves in the formation of TiO_2_ nanoparticles, predominantly located at the Ti_3_C_2_T*_x_* MXene flake edges but on local areas of the basal plane, as schematically represented in Figure 4b. This oxidation mechanism can either be leveraged to prepare Ti_3_C_2_T*_x_* ‐TiO_2_ composite materials^[^ ^33^ ^]^ or avoided by storing the MXene as dry film while removing intercalated water by vacuum annealing.^[^ ^10^ ^]^

## Conclusion

In this work, in situ STXM is employed to investigate redox reactions that may occur with Ti_3_C_2_T*_x_* MXene flakes in humid and aqueous environment. Our findings reveal that wrinkles, folds, and overlapping regions in MXene flakes serve as primary sites for water entrapment, with water molecules exhibiting up to four different phases depending on the type of the confinement configuration. In humid air, limited interaction between water and the MXene surface groups were evidenced. In contrast, few‐layered Ti_3_C_2_T*_x_* MXene flakes are susceptible to water‐induced oxidation under prolonged exposure in water. Specifically, oxidation occurs non‐uniformly over the flakes and seems to affect particularly the edges of individual few‐layered MXene flakes. The oxidation is enhanced upon cation intercalation, which may interact directly with surface functional groups or enable the co‐intercalation of water molecules. Interestingly, the Ti oxidation was found to be partially reversible upon exposure to an acidic environment via a protonation–deprotonation mechanism of the MXene surface. This highlights that most surface titanium atoms are redox active sites for MXene flakes with fewer than 20 layers. These insights are fundamental to understanding the nature of confined water and the redox properties of surface terminations on MXenes at the nanoscale. Beyond MXenes, similar water phases may be found in clay materials and other 2D materials. Local chemical changes of transition metals may also be observed with layered metal oxides. This work demonstrates that in situ STXM is a promising technique to investigate water confinement and intercalation phenomena at the nanoscale that could be extended to other materials.

## Conflict of Interests

The authors declare no conflict of interest.

## Supporting information



Supporting Information

## Data Availability

The data that support the findings of this study are available from the corresponding author upon reasonable request.
