# Supplementary material for: Water‐Induced Local Redox Reactions on Individual Ti3C2T x MXene Flakes in Aqueous Environment
Source: Angew Chem Int Ed Engl. 2025 Dec 4;65(4):e20508. doi: 10.1002/anie.202520508 (PMC12828464; doi:10.1002/anie.202520508)
Supplement: Supplementary file 1 — Supporting Information [file ANIE-65-e20508-s001.docx]

**Supporting Information:** **Water-induced local redox reactions on individual Ti_3_C_2_T*_x_* MXene flakes in aqueous environment**

Faidra Amargianou^1,2^, Peer Bärmann^1^, Namrata Sharma^1,2^, Mailis Lounasvuori^1^, Andreas Furchner^1^, Ralfy Kenaz^3^, Saptarshi Ghosh^3^, Jan-David Förster^4,5^, Christopher Pöhlker^4^, Markus Weigand^1^, and Tristan Petit^1,^*

^1^ Helmholtz-Zentrum Berlin für Materialien und Energie GmbH, Albert-Einstein-Straße 15, 12489 Berlin, Germany

^2^ Faculty of Mathematics and Natural Sciences, TU-Berlin, Hardenbergstraße 36, 10623 Berlin, Germany

^3^ Racah Institute of Physics, The Hebrew University of Jerusalem, Jerusalem 9190401, Israel

^4^ Multiphase Chemistry Department, Max Planck Institute for Chemistry, Hahn-Meitner-Weg 1, 55128 Mainz, Germany

^5^ Atmospheric Microphysics Department, Leibniz Institute for Tropospheric Research, Leipzig, Germany

*Corresponding author. E-mail: tristan.petit@helmholtz-berlin.de


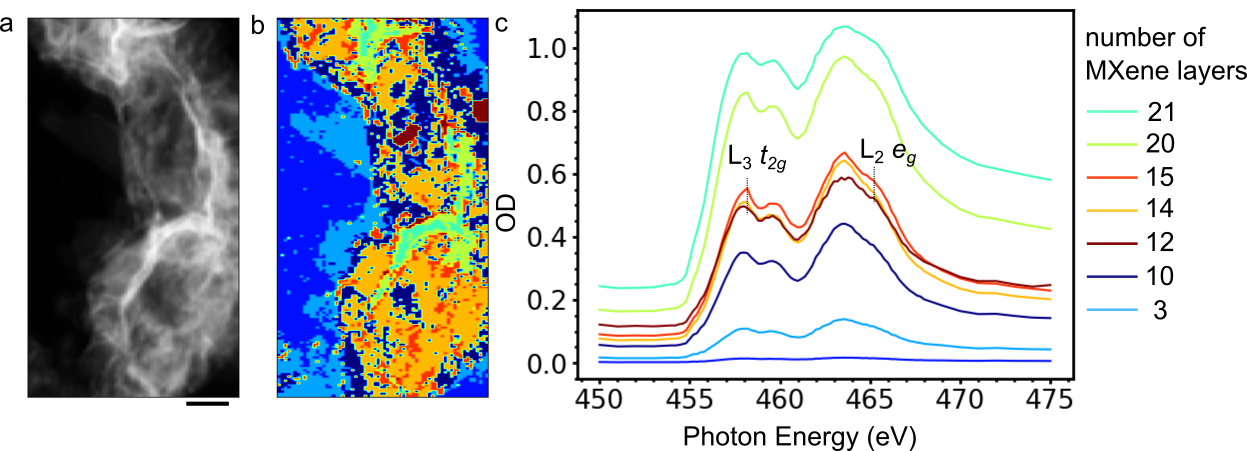


**Fig. S1 Cluster analysis of STXM at Ti L-edge at low humidity. a** STXM image, averaged over the energy range 450-475 eV. **b** Clustered image with principal component followed by gaussian mixture model. **c** Equivalent clustered XAS at the Ti L-edge. The thicker MXene flakes (>12 layers) appear slightly more oxidized, as visible by an increased Ti L_2_ *e_g_* contribution at 465 eV. The thickness of the flakes is estimated by the maximum value of the optical density (OD), as described in reference [1]. Scalebar: 2 μm.


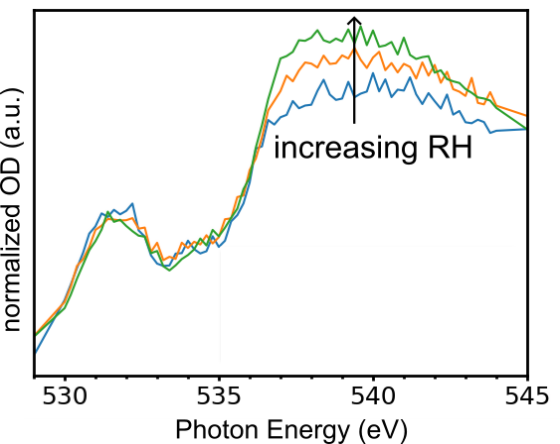


**Fig. S2 Humidity-dependent XAS at the O K-edge.** XAS at O K-edge, averaged over the whole MXene area, for low (5%), intermediate (20%) and high (70%) relative humidity (RH). The increased X-ray absorption in the range 536-550 eV (W_2_ and W_3_) demonstrates the increase of a bulk-like water component.


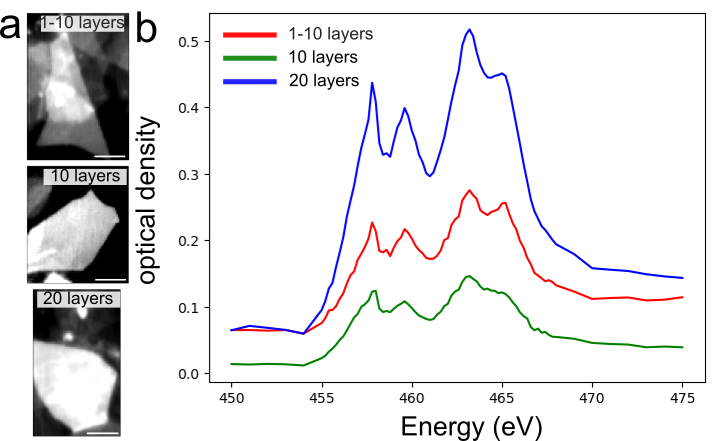


**Fig. S3 a STXM images at the Ti L-edge of Ti_3_C_2_T*_x_*** **MXene flakes** during exposure to N_2_ gas. **b** XAS spectra at the Ti L-edge, averaged over the full area of the flakes. The optical density at XX eV is used to estimate the thickness of the different MXene flakes.


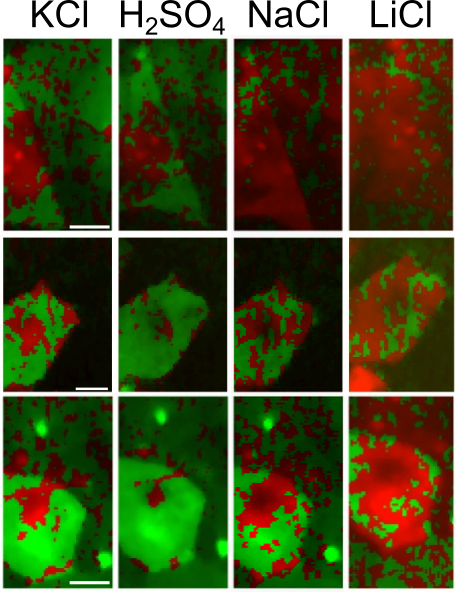


**Fig. S4 in situ STXM images in alkali chloride aqueous electrolytes.** XA images of MXene flakes with thickness 1-10 layers (top), 10 layers (middle) and 20 layers (bottom), during exposure to KCl, H_2_SO_4_, NaCl, H_2_SO_4_ and LiCl. The colors are based on Ti L_2_ *e_g_* to L_2_ *t_2g_* peak intensity ratio, reflecting the oxidation state of Ti atoms, as discussed in the main text. Scale bar: 1 μm.


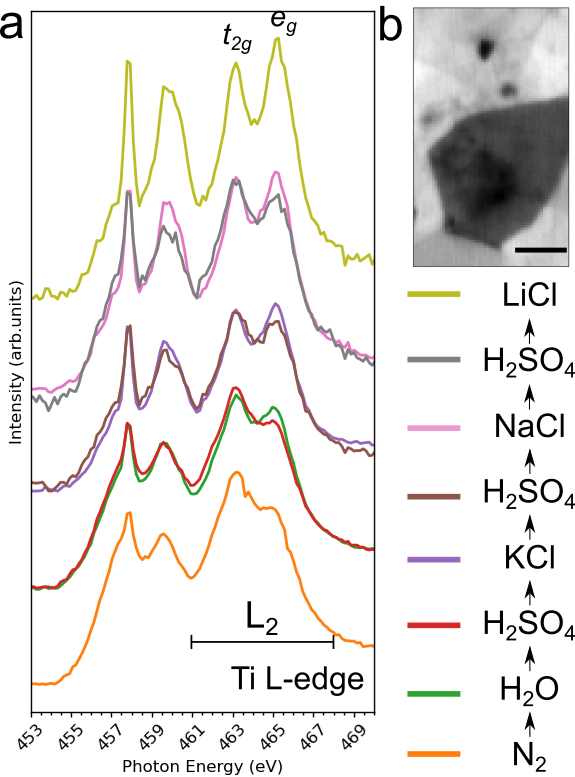


**Fig. S5 Evolution of the Ti chemical bonding in a** **Ti_3_C_2_T*_x_* MXene flake with different aqueous electrolytes. a** XAS spectra at Ti L-edge measured as point scan in the region labelled in Figure 4. The XAS spectra are offset for clarity. **b** STXM image of the 20-layered flakes monitored during sequential exposure to N_2_, H_2_O, H_2_SO_4_, KCl, H_2_SO_4_, NaCl, H_2_SO_4_ and LiCl. Scale bar: 1μm.


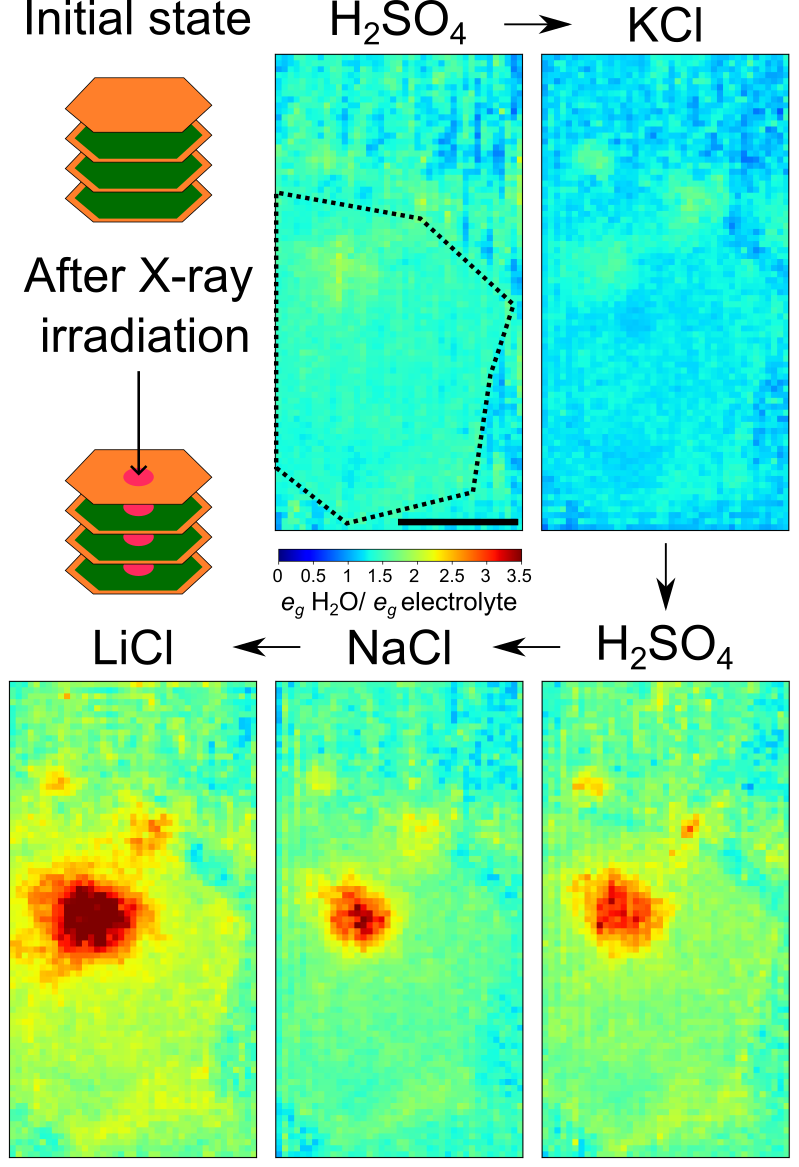


**Fig. S6 X-ray induced local oxidation of a Ti_3_C_2_T*_x_* MXene flake.** Contrast maps between water and aqueous electrolytes of the 20-layered flake. The contrast map is acquired by dividing the STXM image of the flake at Ti L_2_ *e_g_* peak during exposure to water with the STXM image of the flake at the Ti L_2_ *e_g_* peak during exposure to aqueous electrolyte. Scale bar: 1 μm.

**Noise-learning for improved STXM imaging**
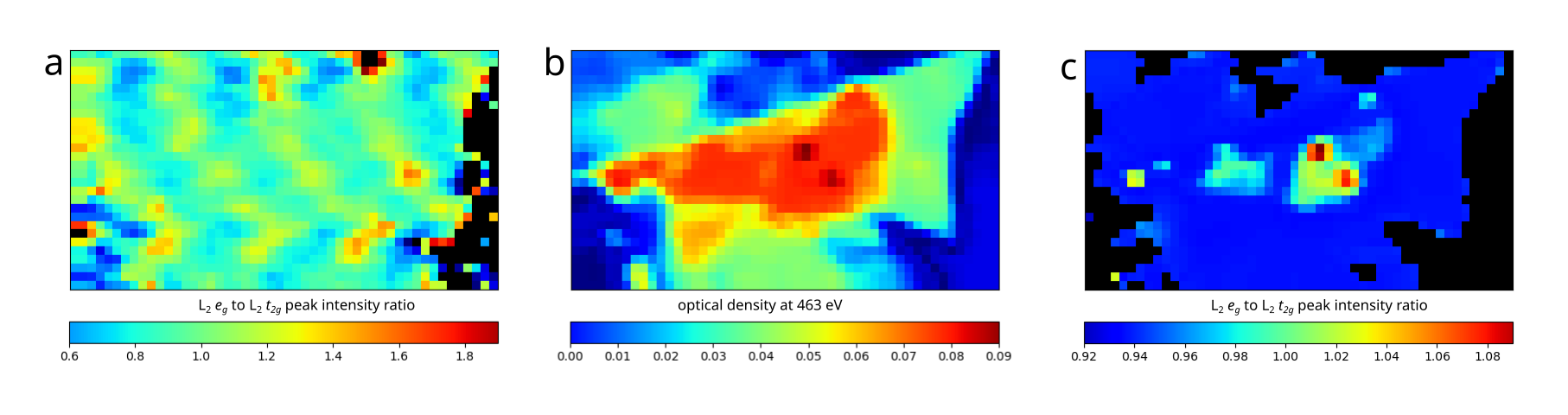


**Fig. S7 in situ STXM denoising for individual MXene flake imaging. a** STXM image of the overlapping flakes at the Ti L_2_ *e_g_* peak divided by the STXM image of the flake at the Ti L_2_ *t_2g_* peak during exposure to N_2_ gas. **b** XA image at 463 eV. **c** Denoised image of the overlapping flakes at L_2_ *e_g_* peak with the image of the flake at the Ti L_2_ *t_2g_* peak.

When the STXM image at 465 eV (maximum of Ti L_2_ e_g_ peak) is divided by the image at 463 eV (maximum of Ti L_2_ t_2g_ peak), mapping the Ti oxidation flake along the flake is hindered by the noise. Fig. S8 a is dominated by the shot noise, characterized by high and low values. If we exclude these values, then the contrast image presents noise related to the STXM instrument (Fig. S8a). The contrast in the STXM image at 463 eV (after subtracting the STXM image at 450 eV) in Fig. S8b depends on the thickness and the absorption coefficient. Even if increased Ti oxidation state corresponds to higher absorption coefficient, Fig. S8b still contains information of the thickness of the flakes. The chemical information can be separated from the thickness information by dividing the denoised STXM image at 465 eV by the STXM image at 463 eV. The artefact is addressed by an autoencoder-based denoising.

The denoising approach uses the following steps:

The 3D cube (X, Y, E) is reshaped into a 2D array of shape (pixels, energy) and normalized to the 99^th^ percentile to reduce sensitivity to outliers. A simple, fully connected neural network is defined, consisting of:

- an encoder that compresses the spectral dimension to a user-defined bottleneck size.
- a decoder that reconstructs the full spectrum from the compressed representation.

Gaussian noise is added to the inputs during training (controlled by the noise_factor parameter) to encourage robustness. The model is trained to reconstruct the clean input spectra using mean squared error (MSE) loss. After training for a specified number of epochs (num_epochs), the trained autoencoder is used to generate denoised spectra, which are then reshaped back to the original (X, Y, E) cube.

The inputs are the following:

cube: Input hyperspectral data cube (np.ndarray) of shape (X, Y, E).

bottleneck: number of neurons in the autoencoder’s bottleneck layer (default: 4).

num_epochs: number of training epochs (default: 300).

noise_factor: standard deviation of Gaussian noise added during training (default: 0.05).

**References**

[1] F. Amargianou *et al.*, “Nanoscale Surface and Bulk Electronic Properties of Ti_3_C_2_T*_x_* MXene Unraveled by Multimodal X-Ray Spectromicroscopy,” *Small Methods*, p. 2400190, 2024.
